# Tendências Temporais e Desfechos Intra-Hospitalares do Implante de Bioprótese Aórtica Transcateter em Valvas Aórticas Bicúspides no Brasil: Uma Análise Pareada por Escore de Propensão

**DOI:** 10.36660/abc.20250750

**Published:** 2026-07-15

**Authors:** Pedro Calomeni, Fernando Bernardi, Fábio Sandoli de Brito, Marcella Sousa Abizaid, Francisco Ribeiro Barbosa, Pedro Alves Lemos, Fausto Feres, Dimytri Alexandre Siqueira, Ricardo Costa, Rogério Sarmento-Leite, Fernanda Mangione, José Armando Mangione, Luiz Eduardo Koenig São Thiago, Valter Correia de Lima, Adriano Dias Dourado Oliveira, Marcos Antônio Marino, Carlos José Francisco Cardoso, Paulo Ricardo Avancini Caramori, Rogério Tadeu Tumelero, Antenor Lages Fortes Portela, Maurício Lopes Prudente, Leônidas Alvarenga Henriques, Fábio Solano de Freitas Souza, Cristiano Guedes Bezerra, Guy Fernandes de Almeida Prado, Leandro Zacarias Figueiredo de Freitas, Ederlon Ferreira Nogueira, George César Ximenes Meireles, Renato Bastos Pope, Ênio Eduardo Guérios, Pedro Beraldo de Andrade, Luciano de Moura Santos, Augusto Celso Lopes, Vinicius Borges Cardozo Esteves, Cleverson Zukowski, Alexandre Abizaid, Henrique Barbosa Ribeiro

**Affiliations:** 1 Universidade de São Paulo Instituto do Coração Faculdade de Medicina São Paulo SP Brasil Instituto do Coração do Hospital das Clínicas da Faculdade de Medicina da Universidade de São Paulo, São Paulo, SP – Brasil; 2 Hospital Israelita Albert Einstein São Paulo SP Brasil Hospital Israelita Albert Einstein, São Paulo, SP – Brasil; 3 Instituto Dante Pazzanese de Cardiologia São Paulo SP Brasil Instituto Dante Pazzanese de Cardiologia, São Paulo, SP – Brasil; 4 Instituto de Cardiologia de Porto Alegre Porto Alegre RS Brasil Instituto de Cardiologia de Porto Alegre, Porto Alegre, RS – Brasil; 5 Beneficência Portuguesa de São Paulo São Paulo SP Brasil Beneficência Portuguesa de São Paulo, São Paulo, SP – Brasil; 6 SOS Cardio Florianópolis SC Brasil SOS Cardio, Florianópolis, SC – Brasil; 7 Santa Casa de Misericórdia de Porto Alegre Porto Alegre RS Brasil Santa Casa de Misericórdia de Porto Alegre, Porto Alegre, RS – Brasil; 8 Hospital Santa Izabel Salvador BA Brasil Hospital Santa Izabel, Salvador, BA – Brasil; 9 Hospital Madre Tereza Belo Horizonte MG Brasil Hospital Madre Tereza, Belo Horizonte, MG – Brasil; 10 Hospital Naval Marcílio Dias Rio de Janeiro RJ Brasil Hospital Naval Marcílio Dias, Rio de Janeiro, RJ – Brasil; 11 Hospital São Lucas da PUCRS Porto Alegre RS Brasil Hospital São Lucas da PUCRS, Porto Alegre, RS – Brasil; 12 Associação Hospitalar Beneficente São Vicente de Paulo Passo Fundo RS Brasil Associação Hospitalar Beneficente São Vicente de Paulo, Passo Fundo, RS – Brasil; 13 Associação Piauiense de Combate ao Câncer Teresina PI Brasil Associação Piauiense de Combate ao Câncer, Teresina, PI – Brasil; 14 Hospital Encore Aparecida de Goiânia GO Brasil Hospital Encore, Aparecida de Goiânia, GO – Brasil; 15 Hospital Albert Sabin Juiz de Fora MG Brasil Hospital Albert Sabin, Juiz de Fora, MG – Brasil; 16 Hospital Cárdio-Pulmonar Salvador BA Brasil Hospital Cárdio-Pulmonar, Salvador, BA – Brasil; 17 Hospital Aliança Rede D’Or Salvador BA Brasil Hospital Aliança Rede D’Or, Salvador, BA – Brasil; 18 Hospital do Coração de Goiás Goiânia GO Brasil Hospital do Coração de Goiás, Goiânia, GO – Brasil; 19 Hospital do Coração de Londrina Londrina PR Brasil Hospital do Coração de Londrina, Londrina, PR – Brasil; 20 Hospital do Servidor Público do Estado IAMSPE São Paulo SP Brasil Hospital do Servidor Público do Estado IAMSPE, São Paulo, SP – Brasil; 21 Hospital Hans Dieter Schmidt Joinville SC Brasil Hospital Hans Dieter Schmidt, Joinville, SC – Brasil; 22 Hospital Nossa Senhora do Pilar Curitiba PR Brasil Hospital Nossa Senhora do Pilar, Curitiba, PR – Brasil; 23 Irmandade da Santa Casa de Misericórdia de Marília Marília SP Brasil Irmandade da Santa Casa de Misericórdia de Marília, Marília, SP – Brasil; 24 Hospital Santa Lúcia Brasília DF Brasil Hospital Santa Lúcia, Brasília, DF – Brasil; 25 Hospital Regional Unimed Fortaleza CE Brasil Hospital Regional Unimed, Fortaleza, CE – Brasil; 26 Rede D’Or São Paulo São Paulo SP Brasil Rede D’Or São Paulo, São Paulo, SP – Brasil

**Keywords:** Estenose da Valva Aórtica, Substituição da Valva Aórtica Transcateter, Implante de Prótese de Valva Cardíaca, Doença da Valva Aórtica Bicúspide

## Abstract

**Fundamento:**

A valva aórtica bicúspide (VAB) é um fator de risco importante para estenose aórtica. No entanto, os desfechos do implante de bioprótese aórtica transcateter (TAVI) permanecem pouco claros, já que a maioria dos ensaios clínicos randomizados excluiu esses pacientes. Além disso, grande parte das evidências publicadas sobre o TAVI em VAB advém de países de alta renda, o que pode não refletir a realidade de países em desenvolvimento.

**Objetivos:**

Avaliar tendências temporais, características do procedimento e desfechos intra-hospitalares entre pacientes com VAB e valva aórtica tricúspide (VAT).

**Métodos:**

Estudo observacional retrospectivo baseado em um registro multicêntrico brasileiro de pacientes submetidos ao TAVI entre janeiro de 2009 e dezembro de 2021. Realizamos pareamento por escore de propensão para ajustar as comparações. Um valor de p<0,05 foi considerado estatisticamente significativo para todas as análises.

**Resultados:**

Entre 2426 pacientes de 25 centros, 111 tinham VAB. Os procedimentos de TAVI aumentaram ao longo do tempo em ambos os grupos. Após o pareamento na proporção de um para três, foram incluídos 90 pacientes com VAB e 243 com VAT. Não foram observadas diferenças significativas nas taxas de complicações vasculares maiores (4% vs. 5%; p=0,99), sangramento maior ou com risco de vida (10% vs. 6%; p=0,34), acidente vascular cerebral (AVC) (2% vs. 0,4%; p=0,37) ou mortalidade intra-hospitalar (7% vs. 3%; p=0,21). Ao longo do tempo, houve redução significativa na mortalidade intra-hospitalar (p<0,01) e nas principais complicações do procedimento (p<0,01 para eventos vasculares, sangramento e AVC), independentemente do tipo de valva.

**Conclusão:**

Durante o período do estudo, os procedimentos anuais de TAVI aumentaram tanto em pacientes com VAB quanto com VAT, embora a prevalência de VAB tenha permanecido relativamente baixa. Não houve diferenças significativas nos desfechos do procedimento ou clínicos entre os grupos pareados por escore de propensão, com redução das complicações do procedimento e da mortalidade para ambos os tipos de valva ao longo do tempo.

**Figure:**
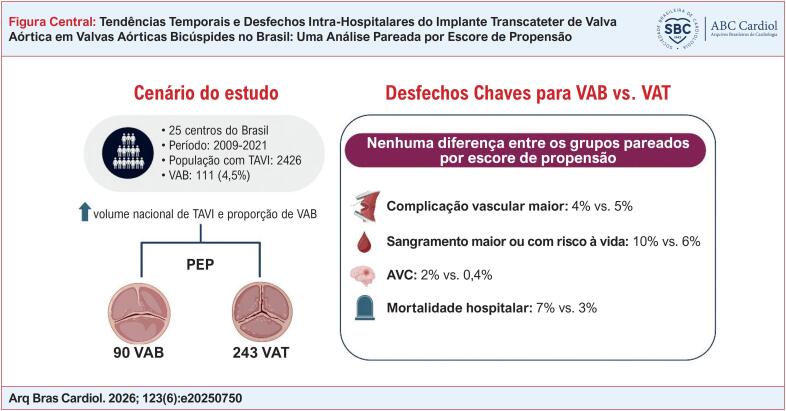
VAB: valva aórtica bicúspide; VAT: valva aórtica tricúspide; PEP: pareamento por escore de propensão; AVC: acidente vascular cerebral.

## Introdução

O implante de bioprótese aórtica transcateter (TAVI, do inglês *transcatheter aortic valve implantation*) é um tratamento bem estabelecido para o manejo da estenose aórtica grave em todo o espectro de risco cirúrgico.^1-4^ A valva aórtica bicúspide (VAB) corresponde a uma proporção significativa dos pacientes com estenose aórtica (EA) submetidos a tratamento valvar. Uma análise de 200 849 adultos que passaram por substituição valvar aórtica cirúrgica por EA grave entre 2011 e 2022, no banco de dados de cirurgia cardíaca da *Society of Thoracic Surgeons* (STS), demonstrou que a prevalência de VAB foi de 27%.^5^ Em contraste, o banco de dados STS TAVI relatou uma prevalência de VAB de aproximadamente 3%.^6^

Apesar de sua relevância clínica, as evidências sobre os desfechos do TAVI em pacientes com VAB permanecem limitadas, uma vez que a maioria dos ensaios clínicos randomizados excluiu essa população.^7^ Além disso, a maior parte dos dados publicados sobre o TAVI em pacientes com VAB origina-se de estudos observacionais conduzidos em países de alta renda, o que pode não refletir adequadamente a realidade de países em desenvolvimento. Há ainda heterogeneidade adicional decorrente de diferenças nas características dos pacientes, nos dispositivos disponíveis, da expertise dos centros e da geração das biopróteses aórticas transcateter (THVs, do inglês transcatheter heart valves) especialmente relevante considerando que as THV de nova geração demonstraram melhor desempenho valvar e maior sucesso do procedimento.^8^

Portanto, os objetivos deste estudo foram: I) avaliar as tendências temporais dos procedimentos de TAVI em pacientes com VAB versus valva aórtica tricúspide (VAT) com EA grave submetidos ao TAVI no Brasil; e II) comparar os desfechos do procedimento e intra-hospitalares entre os grupos VAB e VAT.

## Métodos

### Amostra e protocolo do estudo

Este é um estudo observacional retrospectivo baseado em um registro clínico multicêntrico brasileiro, avaliando variáveis clínicas e de imagem relacionadas ao TAVI entre janeiro de 2009 e dezembro de 2021. Todos os centros no Brasil que realizam TAVI rotineiramente foram contatados e convidados a participar do Registro Brasileiro para Avaliação dos Resultados do Implante de Bioprótese Aórtica por Cateter (RIBAC-NT), coordenado pela Sociedade Brasileira de Hemodinâmica e Cardiologia Intervencionista (SBHCI). Os únicos critérios de exclusão para este estudo foram: procedimentos de valve-in-valve e a ausência de informação sobre a morfologia valvar.

### Coleta de dados

As seguintes informações foram coletadas para cada paciente: (I) características clínicas basais e exames complementares; (II) dados do planejamento do TAVI; (III) técnicas e materiais utilizados no procedimento; (IV) complicações do procedimento; (V) dados relacionados ao cuidado intra-hospitalar após o TAVI.

Os desfechos clínicos foram avaliados de acordo com os critérios VARC-2.^9^ As THVs foram classificadas por tipo de dispositivo e geração da seguinte forma: as biopróteses balão-expansíveis de nova geração incluíram Sapien 3 e Sapien 3 Ultra (Edwards Lifesciences, Irvine, CA, EUA) e Myval (Meril Life Sciences, Vapi, Índia). As biopróteses balão-expansíveis de geração anterior incluíram a Sapien XT (Edwards Lifesciences, Irvine, CA, EUA) e Inovare (Braile Biomédica, São José do Rio Preto, Brasil). As biopróteses autoexpansíveis de nova geração incluíram Evolut R e Evolut PRO (Medtronic, Minneapolis, MN, EUA), Acurate neo e neo 2 (Boston Scientific, Boston, MA, EUA) e Portico (Abbott, Santa Clara, CA, EUA). As biopróteses autoexpansíveis de geração anterior foram representadas exclusivamente pela CoreValve (Medtronic, Minneapolis, MN, EUA). A Lotus Valve (Boston Scientific, Boston, MA, EUA) foi classificada como uma THV expansível mecanicamente.

### Desfechos do estudo

Os desfechos de interesse foram: I) o número anual de procedimentos de TAVI realizados em pacientes com VAB e VAT; II) a proporção de procedimentos realizados em centros de alto volume (volume total ?120 casos);^10^ III) desfechos do procedimento, incluindo implante de marca-passo permanente (IMP), ruptura do anel e oclusão coronariana; IV) desfechos intra-hospitalares para pacientes com VAB vs. VAT, particularmente as taxas de complicações vasculares maiores, sangramento maior ou com risco de vida, acidente vascular cerebral (AVC), mortalidade intra-hospitalar e segurança precoce intra-hospitalar.

### Análise estatística

As variáveis categóricas são apresentadas como frequências absolutas (n) e relativas (%). As variáveis contínuas são expressas como média ± desvio padrão ou mediana [intervalo interquartil], conforme apropriado. A normalidade foi avaliada pelo teste de Anderson–Darling. As comparações das variáveis basais entre os grupos foram realizadas utilizando o teste t de Welch ou o teste de Mann-Whitney para variáveis contínuas, conforme apropriado, e o teste do qui-quadrado para variáveis categóricas.

A proporção anual de casos de VAB em relação à VAT foi calculada, e uma curva de regressão polinomial local foi ajustada para modelar e visualizar as tendências temporais. Para ajustar diferenças intergrupos entre VAB e VAT, as análises foram restritas aos pacientes tratados a partir de 2017, incluindo assim apenas anos com representação significativa de casos VAB (n ? 10). Dentro dessa coorte restrita, realizou-se posteriormente o pareamento por escore de propensão utilizando uma razão de 1:3 com algoritmo *greedy nearest-neighbor*. A lista de variáveis utilizadas no pareamento por escore de propensão encontra-se na Tabela Suplementar 1.

As diferenças das médias padronizadas (SMD) foram utilizadas para avaliar o balanceamento entre todas as variáveis, enquanto as razões de variância (RV) foram calculadas apenas para variáveis contínuas. Considerou-se adequado o balanceamento quando as diferenças das médias padronizadas fossem < 0,1 e RV entre 0,5 e 2.^11,12^ Modelos de regressão logística com o tempo modelado como variável contínua, utilizando *restricted cubic splines* com três nós, foram ajustados para avaliar o efeito do tempo sobre as complicações do procedimento.

Todos os valores de p foram bicaudais, e p<0,05 foi considerado estatisticamente significativo. As análises foram realizadas utilizando o software R, versão 4.5.1 (R Foundation for Statistical Computing, Viena, Áustria).

## Resultados

### Tendências temporais e distribuição geográfica

A Figura Central resume o fluxograma da amostra do estudo para a comparação entre VAB e VAT. Um total de 3194 pacientes foi incluído no registro RIBAC-NT, dos quais 631 não apresentavam informações sobre a morfologia valvar e 137 foram submetidos a procedimentos *valve-in-valve* ou *TAVI-in-TAV*I. Assim, a coorte final do estudo foi composta por 2426 pacientes provenientes de 25 centros.

Houve 10 centros de alto volume, sendo sete localizados na região sudeste, dois na região sul e um na região nordeste, representando conjuntamente 72% da população do estudo.

**Figura 1:**
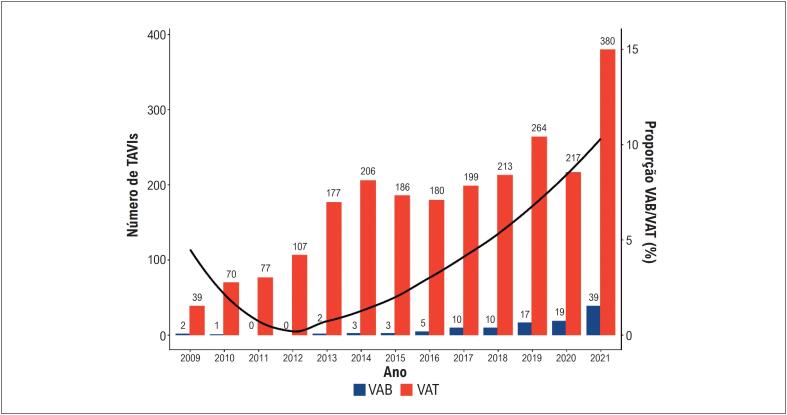
Tendência temporal de pacientes com valva aórtica bicúspide (VAB) e valva aórtica tricúspide (VAT) submetidos ao implante de bioprótese aórtica transcateter (TAVI) no Brasil de 2009 a 2021; a linha preta representa a regressão polinomial local para a proporção VAB/VAT.

O número anual de procedimentos de TAVI realizados no Brasil, estratificado pela morfologia valvar, é apresentado na Figura 1. Ao longo do período do estudo, observou-se um aumento significativo no volume anual de procedimentos de TAVI tanto para pacientes com VAB quanto com VAT. A proporção de procedimentos VAB em relação aos VAT também aumentou ao longo do tempo. A maioria dos procedimentos concentrou-se nas regiões Sudeste e Sul.

### Características da amostra

As características clínicas e ecocardiográficas basais da população do estudo antes e depois do pareamento são apresentadas na Tabela 1. Antes do pareamento, os pacientes com VAB eram mais jovens do que aqueles com VAT, apresentavam menor classe funcional NYHA III/IV e tinham menor carga de comorbidades coronarianas, como doença arterial coronariana prévia e intervenção coronária percutânea. O grupo VAB também apresentou menor EuroSCORE II.

**Tabela 1:** Características clínicas e ecocardiográficas basais de pacientes com valva aórtica bicúspide versus valva aórtica tricúspide submetidos ao TAVI no Brasil, antes e depois do pareamento por escore de propensão

		Não pareado			Pareado		
		Bicúspide (n=111)	Tricúspide (n=2315)	Valor p	Bicúspide (n=90)	Tricúspide (n=243)	Valor p
**Idade, anos**		79,0 [71,0 – 84,1]	82,0 [78,0 – 86,0]	<0,01	78,0 [72,0 – 84,0]	80,0 [74,5 – 84,0]	0,31
**Sexo feminino**		51 (45,9)	1166 (50,4)	0,42	38 (42,2)	106 (43,6)	0,92
**IMC, kg/m^2^**		26,0 ± 5,1	26,5 ± 4,7	0,36	26,6 ± 5,1	26,3 ± 4,2	0,58
**Hipertensão**		86 (77,5)	1868 (80,7)	0,48	71 (78,9)	190 (78,2)	0,99
**Diabetes mellitus**		38 (34,2)	816 (35,2)	0,91	34 (37,8)	93 (38,3)	0,99
**NYHA III/IV**		70 (63,1)	1680 (72,6)	0,04	57 (63,3)	147 (60,5)	0,73
**Fibrilação atrial**		8 (7,2)	288 (12,4)	0,27	6 (6,7)	14 (5,8)	0,86
**DAC**		44 (39,6)	1227 (53,0)	0,01	38 (42,2)	112 (46,1)	0,61
**IM prévio**		5 (4,5)	312 (13,5)	0,01	4 (4,4)	18 (7,4)	0,47
**ICP prévia**		21 (18,9)	662 (28,6)	0,04	18 (20,0)	61 (25,1)	0,41
**CRM prévia**		12 (10,8)	343 (14,8)	0,30	12 (13,3)	31 (12,8)	0,99
**DPOC**		16 (14,4)	410 (17,7)	0,44	13 (14,4)	34 (14,0)	0,99
**Creatinina, mg/dl**		1,1 ± 0,5	1,3 ± 0,9	0,03	1,1 ± 0,5	1,1 ± 0,6	0,82
**Hemoglobina, g/dl**		11,3 ± 2,2	10,8 ± 2,1	0,04	11,2 ± 2,2	11,1 ± 2,0	0,63
**EuroSCORE II, %**		3,0 [1,6 – 6,0]	4,8 [2,7 – 8,5]	<0,01	2,7 [1,5 – 5,9]	3,4 [2,0 – 6,3]	0,15
Centro de alto volume*		63 (56,8)	1695 (73,2)	<0,01	47 (52,2)	129 (53,1)	0,99
**Região**		-	-	<0,01	-	-	0,98
	Centro-Oeste	14 (12,6)	65 (2,8)	-	11 (12,2)	26 (10,7)	-
	Nordeste	14 (12,6)	181 (7,8)	-	12 (13,3)	35 (14,4)	-
	Sul	19 (17,1)	747 (32,3)	-	18 (20,0)	50 (20,6)	-
	Sudeste	64 (57,7)	1322 (57,1)	-	49 (54,4)	132 (54,3)	-
**FEVE, %**		62,0 [51,0 – 68,0]	63,0 [52,0 – 68,0]	0,55	62,0 [50,2 – 68,0]	61,0 [50,0 – 66,0]	0,75
**Gradiente aórtico médio, mmHg**		45,5 ± 19,0	45,6 ± 17,7	0,94	43,3 ± 18,4	42,8 ± 18,2	0,83
**AVA, cm^2^**		0,7 ± 0,2	0,7 ± 0,3	0,73	0,7 ± 0,2	0,8 ± 0,3	0,11
**PSAP**		41,0 ± 13,1	41,9 ± 13,3	0,57	39,7 ± 13,3	39,5 ± 12,4	0,90

Variáveis categóricas são apresentadas como n (%). Variáveis contínuas são expressas como média ± desvio-padrão ou mediana [intervalo interquartil], conforme apropriado; IMC: índice de massa corporal; NYHA: New York Heart Association; DAC: doença arterial coronariana; IM: infarto do miocárdio; ICP: intervenção coronária percutânea; CRM: cirurgia de revascularização do miocárdio; DPOC: doença pulmonar obstrutiva crônica; FEVE: fração de ejeção do ventrículo esquerdo; PSAP: pressão sistólica da artéria pulmonar; centros de alto volume foram definidos como aqueles com volume total de procedimentos ? 120 pacientes.

Após o pareamento na proporção de um para três, realizado na coorte restrita (2017–2021), não foram observadas diferenças basais significativas entre os grupos. A avaliação completa do balanceamento após o pareamento está disponível na Tabela Suplementar 1.

### Desfechos do procedimento

Os desfechos do procedimento para as coortes não pareada e pareada são apresentados na Tabela 2. Na coorte pareada, as biopróteses balão-expansíveis foram preferencialmente selecionadas tanto para pacientes com VAB quanto com VAT. Em ambos os grupos, as THVs de 26 mm foram as mais utilizadas, seguidas pelas THVs de 29 mm. A valvuloplastia com balão pré-implante foi realizada com frequência significativamente maior no grupo VAB, enquanto não foram observadas diferenças significativas nas taxas de dilatação com balão pós-implante ou nos gradientes aórticos médios pós-procedimento. Da mesma forma, as taxas de IMP foram semelhantes entre os grupos. A oclusão coronariana foi rara, com apenas um evento registrado em cada grupo, e nenhuma ruptura do anel foi observada na coorte pareada.

**Tabela 2:** Características dos procedimentos e desfechos intra-hospitalares de pacientes com valva aórtica bicúspide versus tricúspide submetidos ao TAVI no Brasil, antes e depois do pareamento por escore de propensão

		Não pareado				Pareado	
		Bicúspide (n=111)	Tricúspide (n=2315)	Valor p	Bicúspide (n=90)	Tricúspide (n=243)	Valor p
**Acesso femoral**		110 (99,1)	2200 (95,0)	0,82	90 (100,0)	235 (96,7)	0,55
**Bioprótese balão-expansível**		60 (54,1)	1052 (45,4)	0,03	46 (51,1)	133 (54,7)	0,40
**Prótese de nova geração**		96 (86,5)	1304 (56,3)	<0,01	87 (96,7)	234 (96,3)	0,99
**THV**		-	-	<0,01	-	-	0,87
	Acurate neo/neo2*	8 (7,2)	121 (5,2)	-	8 (8,9)	21 (8,6)	-
	CoreValve*	3 (2,7)	566 (24,4)	-	0 (0,0)	1 (0,4)	-
	Evolut R/Pro*	33 (29,7)	532 (23,0)	-	32 (35,6)	78 (32,1)	-
	Inovare‡	0 (0,0)	35 (1,5)	-	0 (0,0)	0 (0,0)	-
	Lotus†	4 (3,6)	52 (2,2)	-	1 (1,1)	0 (0,0)	-
	Myval‡	1 (0,9)	26 (1,1)	-	1 (1,1)	4 (1,6)	-
	Portico*	2 (1,8)	15 (0,6)	-	2 (2,2)	7 (2,9)	-
	Sapien XT‡	8 (7,2)	352 (15,2)	-	2 (2,2)	7 (2,9)	-
	Sapien 3/Ultra‡	51 (45,9)	604 (26,1)	-	43 (47,8)	121 (49,8)	-
**Tamanho da THV**		-	-	0,02	-	-	0,18
	20	4 (3,6)	40 (1,7)	-	4 (4,4)	11 (4,5)	-
	21,5	0 (0,0)	5 (0,2)	-	0 (0,0)	1 (0,4)	-
	23	22 (19,8)	462 (20,0)	-	19 (21,1)	53 (21,8)	-
	24	0 (0,0)	7 (0,3)	-	0 (0,0)	0 (0,0)	-
	24,5	1 (0,9)	2 (0,1)	-	1 (1,1)	0 (0,0)	-
	25	3 (2,7)	81 (3,5)	-	1 (1,1)	14 (5,8)	-
	26	43 (38,7)	816 (35,2)	-	32 (35,6)	76 (31,3)	-
	27	7 (6,3)	47 (2,0)	-	7 (7,8)	5 (2,1)	-
	27,5	0 (0,0)	10 (0,4)	-	0 (0,0)	2 (0,8)	-
	28	0 (0,0)	10 (0,4)	-	0 (0,0)	0 (0,0)	-
	29	25 (22,5)	647 (27,9)	-	20 (22,2)	63 (25,9)	-
	30	0 (0,0)	5 (0,2)	-	0 (0,0)	0 (0,0)	-
	30,5	0 (0,0)	4 (0,2)	-	0 (0,0)	1 (0,4)	-
	31	0 (0,0)	103 (4,4)	-	0 (0,0)	1 (0,4)	-
	34	6 (5,4)	62 (2,7)	-	6 (6,7)	12 (4,9)	-
**Tamanho médio da THV, mm**		26,3 ± 2,9	26,6 ± 2,8	0,34	26,4 ± 3,1	25,8 ± 4,5	0,30
**Predilatação com balão**		66 (59,5)	915 (39,5)	<0,01	48 (53,3)	93 (38,3)	0,02
**Pós-dilatação com balão**		34 (30,6)	677 (29,3)	0,88	28 (31,1)	65 (26,7)	0,52
**Gradiente aórtico médio pós-procedimento, mmHg**		10,9 ± 6,7	9,9 ± 5,9	0,14	7,5 ± 6,9	6,6 ± 6,6	0,28
**Ruptura do anel**		0 (0,0)	10 (0,4)	0,99	0 (0,0)	0 (0,0)	§
**Oclusão de artéria coronária**		1 (0,9)	11 (0,5)	0,99	1 (1,2)	1 (0,4)	0,99
**Embolização da valva**		3 (2,7)	44 (1,9)	0,80	3 (3,3)	2 (0,8)	0,24
**Conversão para cirurgia aberta**		4 (3,6)	49 (2,1)	0,48	3 (3,3)	3 (1,2)	0,42
**IMP**		8 (7,2)	261 (11,3)	0,24	6 (6,7)	27 (11,1)	0,32
**Acidente vascular cerebral**		2 (1,8)	55 (2,4)	0,94	2 (2,2)	1 (0,4)	0,37
**Complicação vascular maior**		5 (4,5)	164 (7,1)	0,39	4 (4,4)	12 (4,9)	0,99
**Sangramento maior ou com risco de vida**		12 (10,8)	218 (9,4)	0,75	9 (10,0)	15 (6,2)	0,34
**Óbito intra-hospitalar**		7 (6,3)	156 (6,7)	0,99	6 (6,7)	7 (2,9)	0,21
**Segurança precoce intra-hospitalar**		101 (90,9)	1971 (85,1)	0,12	81 (90,0)	223 (91,8)	0,77

Variáveis categóricas são apresentadas como n (%). Variáveis contínuas são expressas como média ± desvio-padrão ou mediana [intervalo interquartil], conforme apropriado; IMP: implante de marca-passo permanente; THV: valva cardíaca transcateter

‡ THV expansível por balão

† THV mecanicamente expansível;

* THV autoexpansível

§ O valor de p não pôde ser calculado devido à ausência de eventos em ambos os grupos.

### Desfechos intra-hospitalares na coorte pareada

Os desfechos intra-hospitalares para as coortes não pareada e pareada são apresentados na Tabela 2. Não houve diferenças relevantes nas complicações vasculares maiores entre os grupos VAB e VAT. Da mesma forma, não foram observadas diferenças estatisticamente significativas no desfecho composto de sangramento maior ou com risco de vida, nem nas taxas de AVC. Por fim, a mortalidade intra-hospitalar e os desfechos de segurança precoce não diferiram entre os grupos.

### Tendências temporais dos desfechos intra-hospitalares

**Figura 2:**
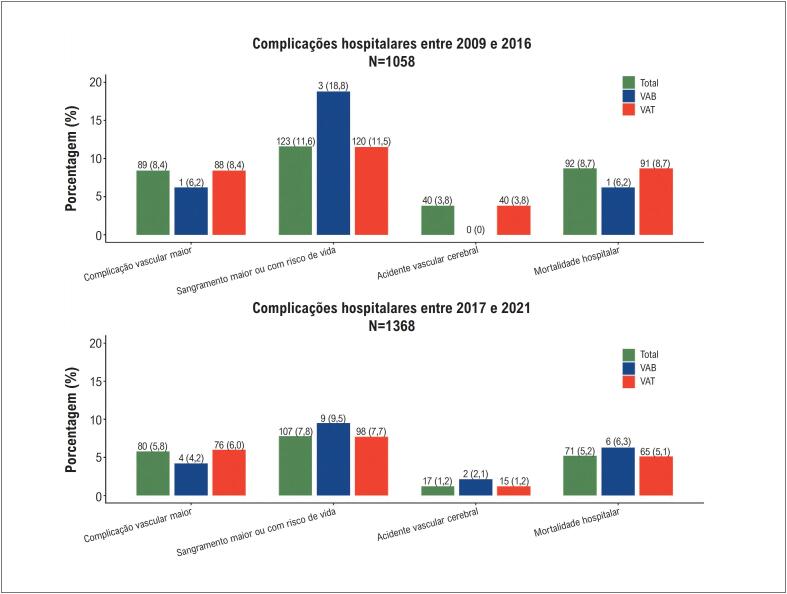
Tendências temporais dos desfechos intra-hospitalares de pacientes submetidos ao implante de bioprótese aórtica transcateter (TAVI) no Brasil; o painel A mostra as taxas de complicações nos primeiros oito anos (2009–2016) do registro RIBAC-NT, enquanto o painel B apresenta as taxas dos últimos cinco anos (2017–2021). VAB: valva aórtica bicúspide; VAT: valva aórtica tricúspide.

A Figura 2 compara os principais desfechos entre os pacientes incluídos nos primeiros oito anos (2009–2016) do registro RIBAC-NT e aqueles dos cinco anos subsequentes (2017–2021). Ao longo do tempo, na coorte geral, todas as complicações do procedimento diminuíram significativamente.

## Discussão

Os principais achados desta análise do registro RIBAC-NT foram: I) durante o período do estudo, houve um aumento significativo no número anual de procedimentos de TAVI realizados tanto em pacientes com VAB quanto com VAT, bem como na razão de volume VAB/VAT; II) as taxas de complicações do TAVI diminuíram ao longo do tempo; III) as complicações vasculares maiores, o desfecho composto de sangramento maior ou com risco de vida, AVC e mortalidade intra-hospitalar não diferiram entre pacientes com VAB e VAT, tanto na coorte geral quanto após o pareamento por escore de propensão.

Apesar do uso crescente do TAVI entre pacientes com EA, poucos estudos avaliaram especificamente sua utilização em indivíduos com VAB. O estudo NOTION-2 foi o único ensaio randomizado que não excluiu pacientes com VAB, e eles representaram apenas 27% da população incluída.^13^ Portanto, os registros atualmente constituem a melhor evidência disponível. Historicamente, pacientes com VAB foram excluídos de ensaios randomizados devido a características anatômicas que podem aumentar o risco de complicações do procedimento ou falha do dispositivo. Essas características incluem anéis aórticos maiores, geometria supra-anular elíptica e calcificação assimétrica extensa – frequentemente com rafe calcificada – que pode dificultar a expansão ideal da THV e aumentar as complicações periprocedimento.^14^

Além disso, pacientes com VAB tendem a ser mais jovens e apresentar menos comorbidades, o que é particularmente relevante diante da incerteza sobre a durabilidade em longo prazo das THVs.^15^ Esses desafios técnicos, combinados com a escassez de evidências provenientes de ensaios randomizados, provavelmente contribuíram para a adoção clínica mais lenta do TAVI em VAB em comparação com VAT, tanto internacionalmente quanto – de forma consistente com nossa análise de tendências temporais – no Brasil.^16,17^

A maior parte das evidências sobre o TAVI em VAB provém de países de alta renda.^14^ No entanto, o contexto brasileiro apresenta desafios específicos, incluindo disparidades substanciais no acesso, diagnóstico tardio e grande variabilidade na experiência dos operadores e centros. Esses fatores influenciam o planejamento do procedimento e seus desfechos, ressaltando a importância de dados reais gerados localmente.^18^ Ainda assim, nossa análise de tendências temporais e a comparação direta entre os resultados de VAT e VAB sustentam a segurança e a eficácia da expansão do TAVI para pacientes selecionados com VAB no Brasil. Assim, o presente estudo pode auxiliar na tomada de decisão clínica em sistemas de saúde com recursos igualmente limitados.

Uma meta-análise prévia sugeriu que pacientes com VAB apresentavam maior risco de AVC, apesar de características basais semelhantes entre as coortes avaliadas.^19^ Esse achado foi atribuído às características anatômicas únicas da VAB, particularmente à presença frequente de calcificação intensa. No entanto, em nosso registro, pacientes com VAB e VAT apresentaram taxas de AVC comparáveis. Isso pode refletir o fato de que a maioria dos procedimentos em nossa coorte foi realizada entre 2017 e 2021, predominantemente com THVs de nova geração. Em contraste, a meta-análise incluiu procedimentos realizados entre 2006 e 2019, com apenas cerca de metade dos pacientes tratados com dispositivos de nova geração. Nossos achados estão alinhados com análises anteriores restritas a THVs de nova geração, que não demonstraram diferenças estatisticamente significativas nas taxas de AVC, bem como com uma análise agrupada que incluiu mais de 30.000 pacientes com VAB.^20-23^

Outra possível explicação para nossos resultados é o viés de seleção. Dada a experiência relativamente limitada dos operadores brasileiros, apenas pacientes com anatomias altamente favoráveis e distribuição de cálcio adequada podem ter sido selecionados para o TAVI. Por fim, nossos achados de taxas comparáveis de complicações vasculares maiores, do composto de sangramento maior ou com risco de vida e de mortalidade intra-hospitalar reforçam ainda mais a literatura existente e sustentam o consenso crescente de que o TAVI pode ser realizado com segurança e eficácia em pacientes selecionados com VAB, utilizando técnicas e tecnologias contemporâneas.

### Limitações

Este estudo apresenta limitações inerentes às diferentes fases de adoção clínica entre os grupos, apesar do uso do pareamento por escore de propensão. Como o TAVI foi inicialmente realizado predominantemente em pacientes com VAT, análises que incluem todo o período do estudo inevitavelmente comparam procedimentos realizados com dispositivos e técnicas de gerações anteriores com aqueles realizados posteriormente, utilizando tecnologia mais avançada. Por outro lado, restringir a análise a anos mais contemporâneos introduz outro desequilíbrio, pois os operadores já haviam acumulado experiência substancial com VAT enquanto ainda estavam na curva de aprendizado para VAB.

Além disso, o uso do pareamento por escore de propensão como estratégia de ajuste reduz inerentemente o tamanho efetivo da amostra e, consequentemente, o poder estatístico das análises. Também não dispúnhamos de granularidade suficiente para distinguir entre diferentes fenótipos de VAB, nem foi possível avaliar o volume e a distribuição do cálcio. Dados ecocardiográficos pós-procedimento abrangentes não estavam disponíveis, o que impediu uma avaliação detalhada do desempenho valvar pós-TAVI e de desfechos como *mismatch* prótese–paciente.

Por fim, como a maior parte dos dados se originou de centros de maior volume na região Sudeste, esses achados podem não ser generalizáveis para centros de volume muito baixo.

## Conclusões

Durante o período do estudo, o número anual de procedimentos de TAVI aumentou significativamente tanto em pacientes com VAB quanto com VAT, embora a prevalência de VAB tenha permanecido relativamente baixa. Não houve diferenças significativas nos desfechos do procedimento ou clínicos entre os grupos pareados por escore de propensão, e tanto pacientes com VAB quanto com VAT apresentaram reduções nas complicações do procedimento e na mortalidade ao longo do tempo.

## Data Availability

Os dados utilizados neste estudo são mantidos pela SBHCI e foram disponibilizados aos autores mediante acordo de uso de dados para a realização desta pesquisa. Por esse motivo, os dados não estão publicamente disponíveis. No entanto, poderão ser disponibilizados mediante solicitação razoável e com a autorização da SBHCI.

## Disponibilidade de Dados

Os dados utilizados neste estudo são mantidos pela SBHCI e foram disponibilizados aos autores mediante acordo de uso de dados para a realização desta pesquisa. Por esse motivo, os dados não estão publicamente disponíveis. No entanto, poderão ser disponibilizados mediante solicitação razoável e com a autorização da SBHCI.

## *Material suplementar

Para informação adicional, por favor, clique aqui.

Material suplementar
