# Supplementary material for: Tendências Temporais e Desfechos Intra-Hospitalares do Implante de Bioprótese Aórtica Transcateter em Valvas Aórticas Bicúspides no Brasil: Uma Análise Pareada por Escore de Propensão
Source: Arq Bras Cardiol. 2026 Jul 15;123(6):e20250750. [Article in Portuguese] doi: 10.36660/abc.20250750 (PMC13399554; doi:10.36660/abc.20250750)
Supplement: Material suplementar [file 0066-782X-abc-123-6-e20250750-Suppl01.pdf]

Tabela Suplementar 1 – Avaliação do balanceamento das covariáveis após o pareamento por escore de propensão para a comparação entre pacientes com válvula aórtica bicúspide e válvula aórtica tricúspide submetidos ao TAVI no Brasil

Variável	Diferença média padronizada	Razão de variâncias
Distância geral das variáveis	0,0019	0,9972
Idade	-0,0125	1,0375
Sexo feminino	-0,0093	-
IMC	0,0570	1,4598
Hipertensão	0,0148	-
Diabetes	-0,0185	-
NYHA	0,0444	-
Fibrilação atrial	0,0074	-
DAC	-0,0167	-
IM prévio	-0,0222	-
ICP prévia	-0,0389	-
CRM prévia	0,0093	-
DPOC	0,0000	-
Creatinina	0,0213	0,8200
Hemoglobina	0,0624	1,2186
Prótese de nova geração (THV)	0,0074	-
FEVE	0,0389	0,9973
Gradiente aórtico médio	0,0248	1,0951
PSAP	0,0184	1,1740
EuroSCORE II	0,0251	1,6760
Centro	0,0296	-
Estado	0,0000	-

"IMC": índice de massa corporal; "CRM": cirurgia de revascularização do miocárdio; "DAC": doença arterial coronariana; "DPOC": doença pulmonar obstrutiva crônica; "FEVE": fração de ejeção do ventrículo esquerdo; "IM": infarto do miocárdio; NYHA: classificação funcional da *New York Heart Association*; PSAP: pressão sistólica da artéria pulmonar; ICP: intervenção coronária percutânea; THV: válvula cardíaca transcateter
