# Supplementary material for: Tendências Temporais e Desfechos Intra-Hospitalares do Implante de Bioprótese Aórtica Transcateter em Valvas Aórticas Bicúspides no Brasil: Uma Análise Pareada por Escore de Propensão
Source: Arq Bras Cardiol. 2026 Jul 15;123(6):e20250750. [Article in Portuguese] doi: 10.36660/abc.20250750 (PMC13399554; doi:10.36660/abc.20250750)
Supplement: Supplemental Materials [file 0066-782X-abc-123-6-e20250750-Suppl01-en.pdf]

Supplemental Table 1 - Covariate balance assessment after propensity score matching for the comparison of bicuspid aortic valve vs. tricuspid aortic valve patients undergoing TAVI in Brazil

Variable	Standardized Mean Difference	Variance Ratio
Overall variable distance	0.0019	0.9972
**Age**	-0.0125	1.0375
**Female gender**	-0.0093	-
**BMI**	0.0570	1.4598
**Hypertension**	0.0148	-
**Diabetes**	-0.0185	-
**NYHA**	0.0444	-
**Atrial fibrillation**	0.0074	-
**CAD**	-0.0167	-
**Previous MI**	-0.0222	-
**Previous PCI**	-0.0389	-
**Previous CABG**	0.0093	-
**COPD**	0.0000	-
**Creatinine**	0.0213	0.8200
**Hemoglobin**	0.0624	1.2186
**New-generation THV**	0.0074	-
**LVEF**	0.0389	0.9973
**Mean aortic gradient**	0.0248	1.0951
**PASP**	0.0184	1.1740
**EuroSCORE II**	0.0251	1.6760
**Center**	0.0296	-
**State**	0.0000	-

BMI: body mass index; CABG: coronary artery bypass grafting; CAD: coronary artery disease; COPD: chronic obstructive pulmonary disease; LVEF: left ventricular ejection fraction; MI: myocardial infarction; NYHA: New York Heart Association functional classification; PASP: pulmonary artery systolic pressure; PCI: percutaneous coronary intervention; THV: transcatheter heart valve
