# Supplementary material for: Tendências Temporais e Desfechos Intra-Hospitalares do Implante de Bioprótese Aórtica Transcateter em Valvas Aórticas Bicúspides no Brasil: Uma Análise Pareada por Escore de Propensão
Source: Arq Bras Cardiol. 2026 Jul 15;123(6):e20250750. [Article in Portuguese] doi: 10.36660/abc.20250750 (PMC13399554; doi:10.36660/abc.20250750)
Supplement: Translation

# Translation: Temporal Trends and In-hospital Outcomes of Transcatheter Aortic Valve Implantation in Bicuspid Aortic Valves in Brazil: A Propensity-Matched Analysis

**DOI:** 10.36660/abc.20250750i

**Authors:** Pedro Calomeni, Fernando Bernardi, Fábio Sandoli de Brito, Marcella Sousa Abizaid, Francisco Ribeiro Barbosa, Pedro Alves Lemos, Fausto Feres, Dimytri Alexandre Siqueira, Ricardo Costa, Rogério Sarmento-Leite, Fernanda Mangione, José Armando Mangione, Luiz Eduardo Koenig São Thiago, Valter Correia de Lima, Adriano Dias Dourado Oliveira, Marcos Antônio Marino, Carlos José Francisco Cardoso, Paulo Ricardo Avancini Caramori, Rogério Tadeu Tumelero, Antenor Lages Fortes Portela, Maurício Lopes Prudente, Leônidas Alvarenga Henriques, Fábio Solano de Freitas Souza, Cristiano Guedes Bezerra, Guy Fernandes de Almeida Prado, Leandro Zacarias Figueiredo de Freitas, Ederlon Ferreira Nogueira, George César Ximenes Meireles, Renato Bastos Pope, Ênio Eduardo Guérios, Pedro Beraldo de Andrade, Luciano de Moura Santos, Augusto Celso Lopes, Vinicius Borges Cardozo Esteves, Cleverson Zukowski, Alexandre Abizaid, Henrique Barbosa Ribeiro

**Affiliations:** 1 São Paulo SP Brazil Instituto do Coração do Hospital das Clínicas da Faculdade de Medicina da Universidade de São Paulo, São Paulo, SP – Brazil; 2 São Paulo SP Brazil Hospital Israelita Albert Einstein, São Paulo, SP – Brazil; 3 São Paulo SP Brazil Instituto Dante Pazzanese de Cardiologia, São Paulo, SP – Brazil; 4 Porto Alegre RS Brazil Instituto de Cardiologia de Porto Alegre, Porto Alegre, RS – Brazil; 5 São Paulo SP Brazil Beneficência Portuguesa de São Paulo, São Paulo, SP – Brazil; 6 Florianópolis SC Brazil SOS Cardio, Florianópolis, SC – Brazil; 7 Porto Alegre RS Brazil Santa Casa de Misericórdia de Porto Alegre, Porto Alegre, RS – Brazil; 8 Salvador BA Brazil Hospital Santa Izabel, Salvador, BA – Brazil; 9 Belo Horizonte MG Brazil Hospital Madre Tereza, Belo Horizonte, MG – Brazil; 10 Rio de Janeiro RJ Brazil Hospital Naval Marcílio Dias, Rio de Janeiro, RJ – Brazil; 11 Porto Alegre RS Brazil Hospital São Lucas da PUCRS, Porto Alegre, RS – Brazil; 12 Passo Fundo RS Brazil Associação Hospitalar Beneficente São Vicente de Paulo, Passo Fundo, RS – Brazil; 13 Teresina PI Brazil Associação Piauiense de Combate ao Câncer, Teresina, PI – Brazil; 14 Aparecida de Goiânia GO Brazil Hospital Encore, Aparecida de Goiânia, GO – Brazil; 15 Juiz de Fora MG Brazil Hospital Albert Sabin, Juiz de Fora, MG – Brazil; 16 Salvador BA Brazil Hospital Cárdio-Pulmonar, Salvador, BA – Brazil; 17 Salvador BA Brazil Hospital Aliança Rede D’Or, Salvador, BA – Brazil; 18 Goiânia GO Brazil Hospital do Coração de Goiás, Goiânia, GO – Brazil; 19 Londrina PR Brazil Hospital do Coração de Londrina, Londrina, PR – Brazil; 20 São Paulo SP Brazil Hospital do Servidor Público do Estado IAMSPE, São Paulo, SP – Brazil; 21 Joinville SC Brazil Hospital Hans Dieter Schmidt, Joinville, SC – Brazil; 22 Curitiba PR Brazil Hospital Nossa Senhora do Pilar, Curitiba, PR – Brazil; 23 Marília SP Brazil Irmandade da Santa Casa de Misericórdia de Marília, Marília, SP – Brazil; 24 Brasília DF Brazil Hospital Santa Lúcia, Brasília, DF – Brazil; 25 Fortaleza CE Brazil Hospital Regional Unimed, Fortaleza, CE – Brazil; 26 São Paulo SP Brazil Rede D’Or São Paulo, São Paulo, SP – Brazil

**Keywords:** Aortic Valve Stenosis, Transcatheter Aortic Valve Replacement, Heart Valve Prosthesis Implantation, Bicuspid Aortic Valve Disease

## Abstract

**Background:**

Bicuspid aortic valve (BAV) is a significant risk factor for aortic stenosis (AS). Yet, the outcomes of transcatheter aortic valve implantation (TAVI) remain unclear, as most randomized trials excluded such patients. Furthermore, most published evidence on TAVI in BAV comes from high-income countries, which may not reflect realities of underserved populations.

**Objectives:**

Evaluate temporal trends, procedural, and in-hospital outcomes between BAV and tricuspid aortic valve (TAV) patients.

**Methods:**

Retrospective observational study based on a Brazilian multicenter registry of TAVI patients between January 2009 and December 2021. We performed propensity score matching to adjust comparisons. A p-value <0.05 was considered statistically significant for all analyses.

**Results:**

Among 2,426 patients from 25 centers, 111 had BAV. TAVI procedures increased over time in both groups. After one-to-three matching, 90 BAV and 243 TAV patients were included. No significant differences were observed in rates of major vascular complications (4% vs. 5%; p=0.99), major or life-threatening bleeding (10% vs. 6%; p=0.34), stroke (2% vs. 0.4%; p=0.37), or in-hospital mortality (7% vs. 3%; p=0.21). Over time, there was a significant reduction in in-hospital mortality (p<0.01) and major procedural complications (p<0.01 for vascular, bleeding, and stroke events), regardless of valve type.

**Conclusion:**

During the study period, annual TAVI procedures increased in both BAV and TAV patients, although the prevalence of BAV remained relatively low. No significant differences were observed in procedural or clinical outcomes between the propensity-matched groups. Both valve types showed reductions in procedural complications and in-hospital mortality over time.

**Figure:** BAV: bicuspid aortic valve; PSM: propensity score matching; TAV: tricuspid aortic valve.

## Introduction

Transcatheter aortic valve implantation (TAVI) is a well-established treatment for managing severe aortic stenosis across the entire surgical risk spectrum.^1-4^ Bicuspid aortic valve (BAV) accounts for a significant proportion of patients with aortic stenosis (AS) undergoing valve treatment. An analysis of 200,849 adults who underwent surgical aortic valve replacement for severe AS between 2011 and 2022 in the Society of Thoracic Surgeons (STS) Cardiac Surgery database demonstrated that the prevalence of BAV was 27%.^5^ In contrast, the STS TAVI database reported a BAV prevalence of approximately 3%.^6^

Despite its clinical relevance, evidence regarding TAVI outcomes in BAV patients remains limited, as most randomized controlled trials have excluded this population.^7^ Furthermore, most published data on TAVI in BAV patients originates from observational studies conducted in high-income countries, which may not accurately represent the realities of underserved populations. Additional heterogeneity stems from variations in patient characteristics, device availability, institutional experience, and transcatheter heart valve (THV) generation, particularly because newer-generation THVs have been associated with improved procedural success and valve performance.^8^

Therefore, the objectives of this study were: I) to evaluate temporal trends in TAVI procedures in BAV vs. tricuspid aortic valve (TAV) patients with severe AS who underwent TAVI in Brazil, and II) to compare procedural and in-hospital outcomes between BAV and TAV groups.

## Methods

### Study sample and protocol

This is a retrospective observational study based on a Brazilian multicenter clinical registry, evaluating clinical and imaging TAVI variables from January 2009 to December 2021. All centers in Brazil that routinely perform TAVI were contacted and invited to participate in the Brazilian Registry for Evaluation of the Results of Aortic Bioprosthesis Catheter Implantation (RIBAC-NT), coordinated by the Brazilian Society of Hemodynamics and Interventional Cardiology (SBHCI). The only exclusion criteria for this study were valve-in-valve procedures and missing valve morphology information.

### Data collection

The following information was collected for each patient: (I) baseline clinical characteristics and complementary tests; (II) TAVI planning data; (III) procedural techniques and materials used; (IV) procedural complications; (V) in-hospital care related to TAVI.

Clinical outcomes were evaluated according to VARC-2 criteria.^9^ THVs were classified by device type and generation as follows: new-generation balloon-expandable valves (BEV) included Sapien 3 and Sapien 3 Ultra (Edwards Lifesciences, Irvine, CA) and Myval (Meril Life Sciences, Vapi, India). Early-generation BEVs included the Sapien XT (Edwards Lifesciences, Irvine, CA) and Inovare (Braile Biomédica, São José do Rio Preto, Brazil). New-generation self-expanding valves (SEV) included Evolut R and Evolut PRO (Medtronic, Minneapolis, MN), Acurate neo/neo 2 (Boston Scientific, Boston, MA) and Portico (Abbott, Santa Clara, CA). Early-generation SEV included CoreValve (Medtronic, Minneapolis, MN). The Lotus Valve (Boston Scientific, Boston, MA) was classified as a mechanically expanded THV.

### Study outcomes

The outcomes of interest were: I) the annual number of TAVI procedures performed in BAV and TAV patients; II) the proportion of procedures performed in high-volume centers (total volume ?120 cases);^10^ III) procedural outcomes, including permanent pacemaker implantation (PPI), annular rupture, and coronary artery occlusion; IV) in-hospital outcomes for BAV vs. TAV patients, particularly the rates of major vascular complications, major or life-threatening bleeding, stroke, in-hospital mortality, and in-hospital early safety.

### Statistical analysis

Categorical variables are reported as absolute (n) and relative (%) frequencies. Continuous variables are expressed as mean ± standard deviation or median [interquartile range], as appropriate. Normality was assessed using the Anderson–Darling test. Comparisons of baseline variables between groups were performed using Welch's t-test or the Mann–Whitney U test for continuous variables, as appropriate, and the chi-square test for categorical variables.

The annual proportion of BAV cases relative to TAV was calculated, and a local polynomial regression curve was fitted to model and visualize temporal trends. To adjust for intergroup differences between BAV and TAV, analyses were restricted to patients treated from 2017 onward, thereby including only years with a meaningful representation of BAV cases (n ? 10). Within this restricted cohort, propensity score matching was subsequently performed using a 1:3 ratio with a greedy nearest-neighbor algorithm. The list of variables used for propensity score matching is provided in Supplemental Table 1.

**Table 1:** Baseline clinical and echocardiographic characteristics of patients with bicuspid aortic valve vs. tricuspid aortic valve undergoing TAVI in Brazil, before and after propensity score matching

		Non-matched			Matched		
		Bicuspid (n=111)	Tricuspid (n=2,315)	p-value	Bicuspid (n=90)	Tricuspid (n=243)	p-value
**Age, y**		79.0 [71.0 – 84.1]	82.0 [78.0 – 86.0]	<0.01	78.0 [72.0 – 84.0]	80.0 [74.5 – 84.0]	0.31
**Female gender**		51 (45.9)	1,166 (50.4)	0.42	38 (42.2)	106 (43.6)	0.92
**BMI, kg/m^2^**		26.0 ± 5.1	26.5 ± 4.7	0.36	26.6 ± 5.1	26.3 ± 4.2	0.58
**Hypertension**		86 (77.5)	1,868 (80.7)	0.48	71 (78.9)	190 (78.2)	0.99
**Diabetes mellitus**		38 (34.2)	816 (35.2)	0.91	34 (37.8)	93 (38.3)	0.99
**NYHA III/IV**		70 (63.1)	1,680 (72.6)	0.04	57 (63.3)	147 (60.5)	0.73
**Atrial fibrillation**		8 (7.2)	288 (12.4)	0.27	6 (6.7)	14 (5.8)	0.86
**CAD**		44 (39.6)	1,227 (53.0)	0.01	38 (42.2)	112 (46.1)	0.61
**Previous MI**		5 (4.5)	312 (13.5)	0.01	4 (4.4)	18 (7.4)	0.47
**Previous PCI**		21 (18.9)	662 (28.6)	0.04	18 (20.0)	61 (25.1)	0.41
**Previous CABG**		12 (10.8)	343 (14.8)	0.30	12 (13.3)	31 (12.8)	0.99
**COPD**		16 (14.4)	410 (17.7)	0.44	13 (14.4)	34 (14.0)	0.99
**Creatinine, mg/dl**		1.1 ± 0.5	1.3 ± 0.9	0.03	1.1 ± 0.5	1.1 ± 0.6	0.82
**Hemoglobin, g/dl**		11.3 ± 2.2	10.8 ± 2.1	0.04	11.2 ± 2.2	11.1 ± 2.0	0.63
**EuroSCORE II, %**		3.0 [1.6 – 6.0]	4.8 [2.7 – 8.5]	<0.01	2.7 [1.5 – 5.9]	3.4 [2.0 – 6.3]	0.15
High-volume center*		63 (56.8)	1,695 (73.2)	<0.01	47 (52.2)	129 (53.1)	0.99
**Region**		-	-	<0.01	-	-	0.98
	Central-West	14 (12.6)	65 (2.8)	-	11 (12.2)	26 (10.7)	-
	Northeast	14 (12.6)	181 (7.8)	-	12 (13.3)	35 (14.4)	-
	South	19 (17.1)	747 (32.3)	-	18 (20.0)	50 (20.6)	-
	Southeast	64 (57.7)	1,322 (57.1)	-	49 (54.4)	132 (54.3)	-
**LVEF, %**		62.0 [51.0 – 68.0]	63.0 [52.0 – 68.0]	0.55	62.0 [50.2 – 68.0]	61.0 [50.0 – 66.0]	0.75
**Mean aortic gradient, mmHg**		45.5 ± 19.0	45.6 ± 17.7	0.94	43.3 ± 18.4	42.8 ± 18.2	0.83
**AVA, cm^2^**		0.7 ± 0.2	0.7 ± 0.3	0.73	0.7 ± 0.2	0.8 ± 0.3	0.11
**PASP, mmHg**		41.0 ± 13.1	41.9 ± 13.3	0.57	39.7 ± 13.3	39.5 ± 12.4	0.90

Categorical variables are reported as n (%). Continuous variables are expressed as mean ± standard deviation or median [interquartile range], as appropriate. AVA: aortic valve area; BMI: body mass index; CABG: coronary artery bypass grafting; CAD: coronary artery disease; COPD: chronic obstructive pulmonary disease; LVEF: left ventricular ejection fraction; MI: myocardial infarction; NYHA: New York Heart Association functional classification; PASP: pulmonary artery systolic pressure; PCI: percutaneous coronary intervention.

* High-volume centers were defined as those with total procedural volume ? 120 patients.

Standardized mean differences (SMD) were used to assess balance across all variables, while variance ratios (VR) were calculated only for continuous variables. Variables were considered adequately balanced if standardized mean differences < 0.1 and VR between 0.5 and 2.^11,12^ Logistic regression models with time modeled as a continuous variable using restricted cubic splines with three knots were fitted to evaluate the effect of time on procedural complications.

All p-values were 2-sided, and p<0.05 was considered statistically significant. Data analysis was performed using R Statistical Software version 4.5.1 (R Foundation for Statistical Computing, Vienna, Austria).

## Results

### Temporal trends and geographical distribution

The Central Illustration summarizes the flow diagram of the study sample for the BAV vs. TAV comparison. A total of 3,194 patients were included in the RIBAC-NT registry, of whom 631 lacked information on valve morphology and 137 underwent valve-in-valve or TAVI-in-TAVI procedures. Therefore, the final study cohort comprised 2,426 patients from 25 centers.

There were 10 high-volume centers, 7 located in the Southeast region, 2 in the South, and 1 in the Northeast, collectively accounting for 72% of the study population.

The annual number of TAVI procedures performed in Brazil, stratified by valve morphology, is shown in Figure 1. Over the study period, a significant increase in the annual volume of TAVI procedures was observed for both BAV and TAV patients. The proportion of BAV relative to TAV procedures also increased over time. Most procedures were concentrated in the Southeast and South regions.

**Figure 1:** Temporal trends of bicuspid aortic valve (BAV) and tricuspid aortic valve (TAV) patients undergoing TAVI in Brazil from 2009 to 2021; the black line shows the local polynomial regression for BAV/TAV proportion.

### Sample characteristics

Baseline clinical and echocardiographic characteristics of the study population before and after matching are shown in Table 1. Before matching, BAV patients were younger than TAV, exhibited lower NYHA III/IV functional class, and presented a lower burden of coronary comorbidities, such as previous coronary artery disease and percutaneous coronary intervention. BAV group also had a lower EuroSCORE II. After one-to-three matching performed in the restricted cohort (2017–2021), no significant baseline differences were observed between groups. The complete matching balance assessment is available in Supplemental Table 1.

### Procedural outcomes

Procedural outcomes for the unmatched and matched cohorts are presented in Table 2. In the matched cohort, balloon-expandable THVs were preferentially selected for both BAV and TAV patients. In both groups, the 26-mm valve was used most frequently, followed by the 29-mm valve. Pre-implant balloon valvuloplasty was performed significantly more often in the BAV group, whereas no significant differences were observed in post-implant balloon dilation rates or in postprocedural mean aortic gradients. Likewise, PPI rates were similar between BAV and TAV patients. Coronary artery occlusion was rare, with only one event recorded in each group, and no annular ruptures were observed in the matched cohort.

### In-hospital outcomes in the matched cohort

In-hospital outcomes for the unmatched and matched cohorts are shown in Table 2. There was no meaningful difference in major vascular complication between the BAV and TAV groups. Similarly, no statistically significant differences were observed in the composite outcome of major or life-threatening bleeding or in the stroke rates. Finally, in-hospital mortality and early safety outcomes did not differ between groups.

**Table 2:** Procedural characteristics and in-hospital outcomes of patients with bicuspid aortic valve vs. tricuspid aortic valve undergoing TAVI in Brazil, before and after propensity score matching

		Non-matched			Matched		
		Bicuspid (n=111)	Tricuspid (n=2,315)	p-value	Bicuspid (n=90)	Tricuspid (n=243)	p-value
**Femoral approach**		110 (99.1)	2,200 (95.0)	0.82	90 (100.0)	235 (96.7)	0.55
**THV type (BEV)**		60 (54.1)	1,052 (45.4)	0.03	46 (51.1)	133 (54.7)	0.40
**New-generation THV**		96 (86.5)	1,304 (56.3)	<0.01	87 (96.7)	234 (96.3)	0.99
**THV**		-	-	<0.01	-	-	0.87
	Acurate neo/neo2*	8 (7.2)	121 (5.2)	-	8 (8.9)	21 (8.6)	-
	CoreValve*	3 (2.7)	566 (24.4)	-	0 (0.0)	1 (0.4)	-
	Evolut R/Pro*	33 (29.7)	532 (23.0)	-	32 (35.6)	78 (32.1)	-
	Inovare‡	0 (0.0)	35 (1.5)	-	0 (0.0)	0 (0.0)	-
	Lotus†	4 (3.6)	52 (2.2)	-	1 (1.1)	0 (0.0)	-
	Myval‡	1 (0.9)	26 (1.1)	-	1 (1.1)	4 (1.6)	-
	Portico*	2 (1.8)	15 (0.6)	-	2 (2.2)	7 (2.9)	-
	Sapien XT‡	8 (7.2)	352 (15.2)	-	2 (2.2)	7 (2.9)	-
	Sapien 3/Ultra‡	51 (45.9)	604 (26.1)	-	43 (47.8)	121 (49.8)	-
**THV size**		-	-	0.02	-	-	0.18
	20	4 (3.6)	40 (1.7)	-	4 (4.4)	11 (4.5)	-
	21.5	0 (0.0)	5 (0.2)	-	0 (0.0)	1 (0.4)	-
	23	22 (19.8)	462 (20.0)	-	19 (21.1)	53 (21.8)	-
	24	0 (0.0)	7 (0.3)	-	0 (0.0)	0 (0.0)	-
	24.5	1 (0.9)	2 (0.1)	-	1 (1.1)	0 (0.0)	-
	25	3 (2.7)	81 (3.5)	-	1 (1.1)	14 (5.8)	-
	26	43 (38.7)	816 (35.2)	-	32 (35.6)	76 (31.3)	-
	27	7 (6.3)	47 (2.0)	-	7 (7.8)	5 (2.1)	-
	27.5	0 (0.0)	10 (0.4)	-	0 (0.0)	2 (0.8)	-
	28	0 (0.0)	10 (0.4)	-	0 (0.0)	0 (0.0)	-
	29	25 (22.5)	647 (27.9)	-	20 (22.2)	63 (25.9)	-
	30	0 (0.0)	5 (0.2)	-	0 (0.0)	0 (0.0)	-
	30.5	0 (0.0)	4 (0.2)	-	0 (0.0)	1 (0.4)	-
	31	0 (0.0)	103 (4.4)	-	0 (0.0)	1 (0.4)	-
	34	6 (5.4)	62 (2.7)	-	6 (6.7)	12 (4.9)	-
**Mean THV size, mm**		26.3 ± 2.9	26.6 ± 2.8	0.34	26.4 ± 3.1	25.8 ± 4.5	0.30
**Balloon predilation**		66 (59.5)	915 (39.5)	<0.01	48 (53.3)	93 (38.3)	0.02
**Balloon postdilation**		34 (30.6)	677 (29.3)	0.88	28 (31.1)	65 (26.7)	0.52
**Postprocedural mean aortic gradient, mmHg**		10.9 ± 6.7	9.9 ± 5.9	0.14	7.5 ± 6.9	6.6 ± 6.6	0.28
**Annular rupture**		0 (0.0)	10 (0.4)	0.99	0 (0.0)	0 (0.0)	§
**Coronary artery occlusion**		1 (0.9)	11 (0.5)	0.99	1 (1.2)	1 (0.4)	0.99
**Valve embolization**		3 (2.7)	44 (1.9)	0.80	3 (3.3)	2 (0.8)	0.24
**Conversion to open surgery**		4 (3.6)	49 (2.1)	0.48	3 (3.3)	3 (1.2)	0.42
**PPI**		8 (7.2)	261 (11.3)	0.24	6 (6.7)	27 (11.1)	0.32
**Stroke**		2 (1.8)	55 (2.4)	0.94	2 (2.2)	1 (0.4)	0.37
**Major vascular complication**		5 (4.5)	164 (7.1)	0.39	4 (4.4)	12 (4.9)	0.99
**Major or life-threatening bleeding**		12 (10.8)	218 (9.4)	0.75	9 (10.0)	15 (6.2)	0.34
**In-hospital death**		7 (6.3)	156 (6.7)	0.99	6 (6.7)	7 (2.9)	0.21
**In-hospital early safety**		101 (90.9)	1,971 (85.1)	0.12	81 (90.0)	223 (91.8)	0.77

Categorical variables are reported as n (%). Continuous variables are expressed as mean ± standard deviation or median [interquartile range], as appropriate. BEV: balloon-expandable valve; PPI: permanent pacemaker implantation; THV: transcatheter heart valve.

‡ Balloon-expandable THV.

† Mechanically expandable THV.

* Self-expanding THV.

§ p-value could not be computed due to zero events in both groups.

### Temporal trends for in-hospital outcomes

Figure 2 compares key outcomes between patients enrolled during the first eight years (2009–2016) of the RIBAC-NT registry and those from the subsequent five years (2017–2021). Over time, in the overall cohort, all procedural complications decreased significantly.

**Figure 2:** Temporal trends of in-hospital outcomes for patients undergoing TAVI in Brazil; panel A shows complication rates for the first eight years (2009–2016) of the RIBAC-NT registry, while panel B presents rates for the last five years (2017–2021). BAV: bicuspid aortic valve; TAV: tricuspid aortic valve.

## Discussion

The main findings of this analysis of the RIBAC-NT registry were as follows: I) during the study period, there was a significant increase in the annual number of TAVI procedures performed in both BAV and TAV patients, as well as in the BAV/TAV volume ratio; II) TAVI procedural complication rates decreased over time; III) major vascular complication, the composite outcome of major or life-threatening bleeding, stroke, and in-hospital mortality did not differ between BAV and TAV patients, both in the overall cohort and after propensity score matching.

Despite the growing use of TAVI among patients with AS, very few studies have evaluated its use specifically in individuals with BAV. The NOTION-2 trial was the only randomized study that did not exclude BAV patients, and they represented only 27% of the enrolled population.^13^ Therefore, registries currently provide the best available evidence. Historically, BAV patients have been excluded from randomized trials due to anatomical features that may increase the risk of procedural complications or device failure. These features include larger aortic annuli, elliptical supra-annular geometry, and extensive asymmetric calcification – often with a calcified raphe – that may hinder optimal THV expansion and increase periprocedural complications.^14^

Additionally, BAV patients tend to be younger and have fewer comorbidities, which is particularly relevant given the uncertainty surrounding the long-term durability of THVs.^15^ These technical challenges, combined with the limited randomized evidence, have likely contributed to the slower clinical adoption of TAVI in BAV compared with TAV, both internationally and – consistent with our temporal trend analysis – in Brazil.^16,17^

Most evidence regarding TAVI in BAV comes from high-income countries.^14^ The Brazilian context, however, presents unique challenges, including substantial disparities in access, delayed diagnosis, and considerable variability in operator and center experience. These factors influence procedural planning and outcomes, underscoring the importance of locally generated real-world data.^18^ Nevertheless, our temporal trend analysis and the direct comparison of TAV and BAV outcomes support the safety and effectiveness of extending TAVI to selected BAV patients in Brazil. Thus, the present study may help inform clinical decision-making in similarly resource-limited healthcare systems.

A previous meta-analysis suggested that BAV patients had an increased risk of stroke, despite similar baseline characteristics across cohorts.^19^ This finding was attributed to the unique anatomical features of BAV, particularly the frequent presence of heavy calcification. In our registry, however, BAV and TAV patients exhibited comparable stroke rates. This may reflect the fact that most procedures in our cohort were performed between 2017 and 2021, predominantly using new-generation THVs. In contrast, the meta-analysis included procedures performed between 2006 and 2019, with only about half of the patients treated with new-generation devices. Our findings align with previous analyses restricted to new-generation THVs, which have shown no statistically significant differences in stroke rates, as well as with pooled evaluations including more than 30,000 BAV patients.^20-23^

Another possible explanation for our results is selection bias. Given the relatively limited experience of Brazilian operators, only patients with highly favorable anatomies and calcium distribution may have been selected for TAVI. Finally, our findings of comparable rates of major vascular complications, the composite of major or life-threatening bleeding, and in-hospital mortality further reinforce existing literature and support the growing consensus that TAVI can be performed safely and effectively in selected BAV patients using contemporary techniques and technologies.

### Limitations

This study has inherent limitations related to the different timelines of clinical adoption between groups, despite the use of propensity score matching. Because TAVI was initially performed predominantly in TAV patients, analyses including the entire study period inevitably compare procedures performed with earlier-generation devices and techniques against those performed later using more advanced technology. Conversely, restricting the analysis to more contemporary years introduces another imbalance, as operators had already accumulated substantial experience with TAV while still being on the learning curve for BAV.

In addition, the use of propensity score matching as an adjustment strategy inherently reduces the effective sample size and, consequently, the statistical power of the analyses. We also lacked sufficient granularity to distinguish among different BAV phenotypes, nor were we able to assess calcium volume and distribution. Comprehensive postprocedural echocardiographic data were unavailable, preventing a detailed evaluation of post-TAVI valve performance and outcomes such as prosthesis–patient mismatch.

Finally, because most data originated from higher-volume centers in the Southeast region, these findings may not be generalizable to very low-volume centers.

## Conclusions

During the study period, the annual number of TAVI procedures increased significantly in both BAV and TAV patients, although the prevalence of BAV remained relatively low. There were no significant differences in procedural or clinical outcomes between the propensity-matched groups, and both BAV and TAV patients experienced reductions in procedural complications and mortality over time.

## Data Availability

The data are held by SBHCI, but restrictions apply to their availability, as they were used under license for the present study. However, data may be obtained upon reasonable request and with permission from SBHCI.

## Availability of Research Data

The data are held by SBHCI, but restrictions apply to their availability, as they were used under license for the present study. However, data may be obtained upon reasonable request and with permission from SBHCI.

## *Supplemental Materials

For additional information, please click here.
